# Supplementary material for: Highly reversible zinc metal anode enabled by strong Brønsted acid and hydrophobic interfacial chemistry
Source: Nat Commun. 2024 May 21;15:4303. doi: 10.1038/s41467-024-48444-5 (PMC11109197; doi:10.1038/s41467-024-48444-5)
Supplement: Supplementary file 1 — Supporting information document [file 41467_2024_48444_MOESM1_ESM.docx]

Supplementary Information

**Highly Reversible Zinc Metal Anode Enabled by Strong Brønsted Acid and Hydrophobic Interfacial Chemistry**

Qingshun Nian, Xuan Luo, Digen Ruan, Yecheng Li, Bing-Qing Xiong, Zhuangzhuang Cui, Zihong Wang, Qi Dong, Jiajia Fan, Jinyu Jiang, Jun Ma, Zhihao Ma, Dazhuang Wang, Xiaodi Ren*

*Hefei National Research Center for Physical Sciences at the Microscale, CAS Key Laboratory of Materials for Energy Conversion, Department of Materials Science and Engineering, University of Science and Technology of China, Hefei, Anhui 230026, China

E-mail: [xdren@ustc.edu.cn](mailto:xdren@ustc.edu.cn)


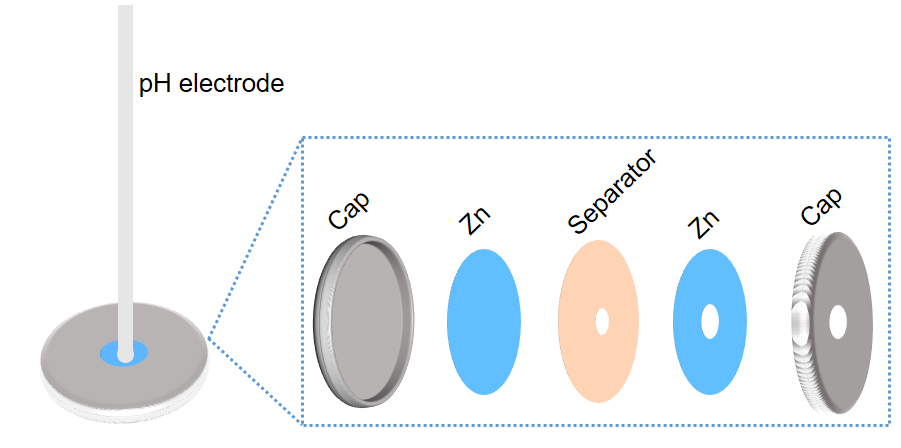


**Supplementary Fig. 1 | Schematic diagram of the home-made *in situ* pH detection configuration.** A pH monitoring setup was constructed to monitor the pH change at the Zn anode in real-time. A specific pH detector for solid interface detection was connected to a Zn anode into which a fixed volume (100 µL) of aqueous electrolyte was dripped. This can help us monitor the actual pH change of the Zn anode as accurately as possible and study the pH change caused by self-corrosion.


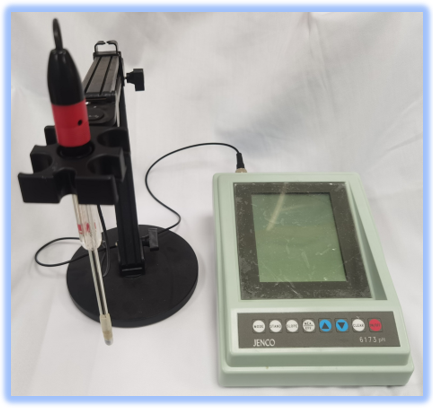
**Supplementary Fig. 2 | Photo of the pH meter**. A device designed with microcomputer functionality and equipped with the STMirco5 pH electrode (ɸ 5 mm) for precise pH measurements. The in-situ pH data were automatically logged by a computer system.


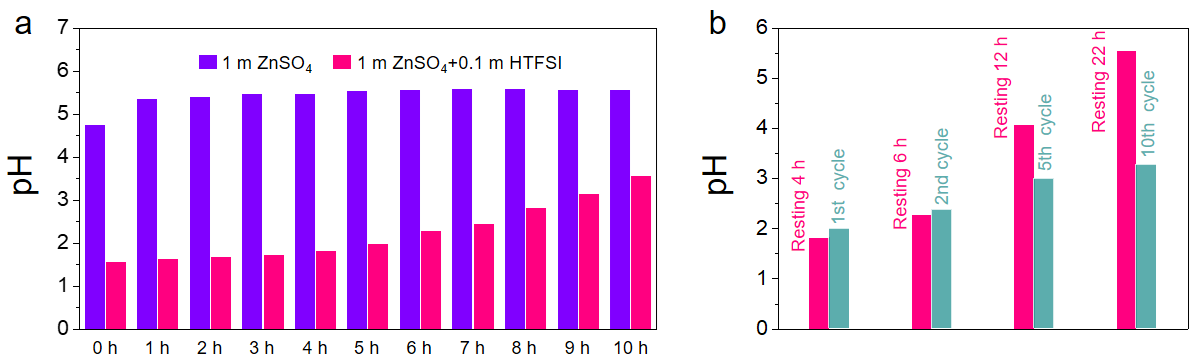


**Supplementary Fig. 3 | pH evolution after battery resting and cycling.** a) Interfacial pH evolution of Zn/Zn symmetrical cell after resting for 0-10 h; b) pH comparison during resting and cycling.


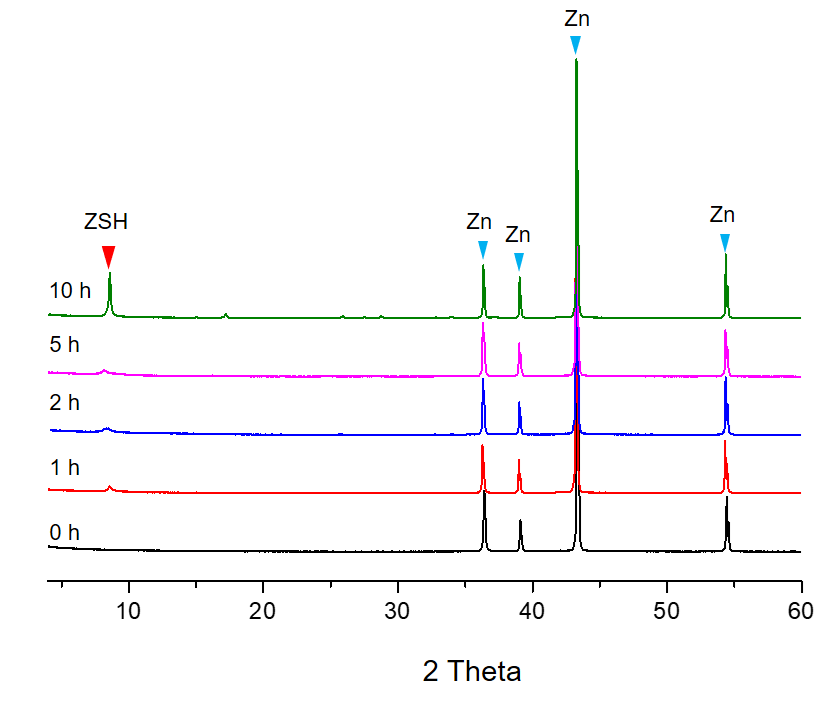


**Supplementary Fig. 4| XRD characterization.** XRD patterns of Zn foil soaked in 1 m ZnSO_4_ electrolytes for different times. Before soaking, the Zn foils were cleaned with sandpaper to remove the passivation layer on the surface.


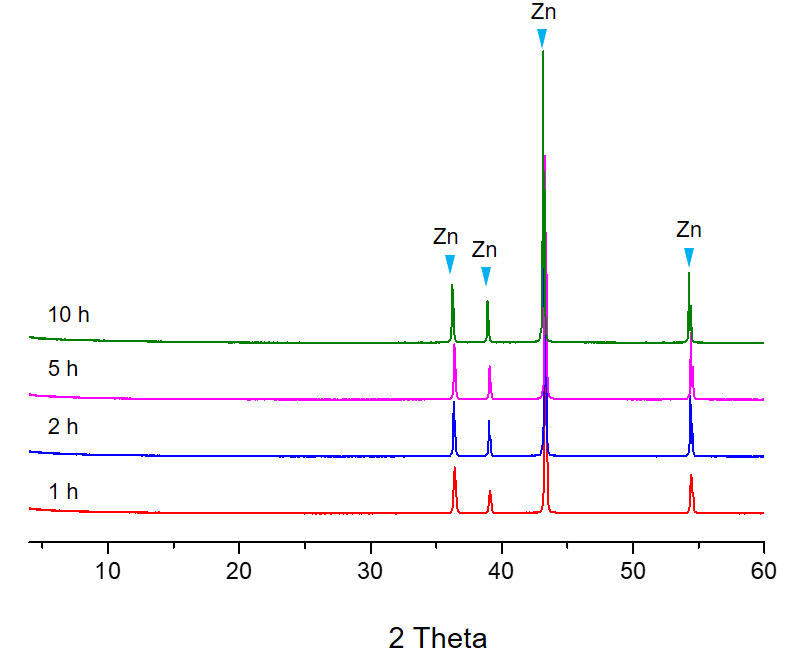


**Supplementary Fig. 5 | XRD characterization.** XRD patterns of Zn foil soaked in 1 m ZnSO_4_ + 0.1 m HTFSI electrolytes for different times. Before soaking, the Zn foils were cleaned with sandpaper to remove the passivation layer on the surface.


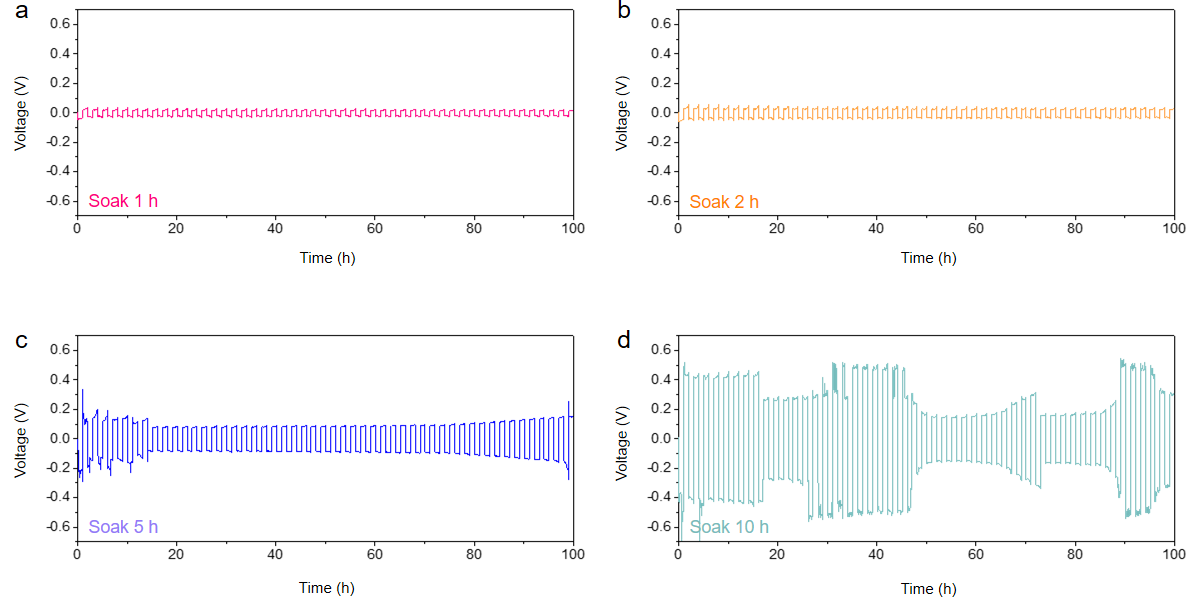


**Supplementary Fig. 6 | Voltage-time curves of Zn||Zn cells under 1 mA cm^−2^, 1 mAh cm^−2^.** The Zn electrodes were soaked in 1 m ZnSO_4_ electrolyte for different times before use. **a** soaking 1 h; **b** soaking 2 h; **c** soaking 5 h; **d** soaking 10 h.


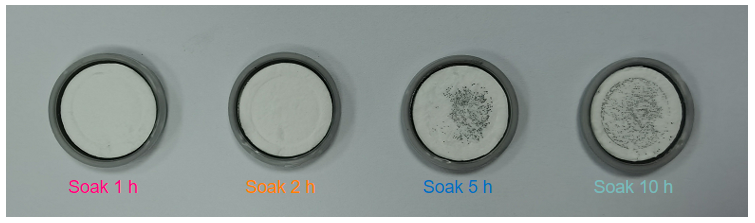


**Supplementary Fig. 7 | Optical images of the separator** after 100 h cycling of the assembled Zn||Zn cell in Supplementary Fig. 4. Batteries assembled with Zn electrodes soaked for 5 h and 10 h showed a large number of dendrites attached to the separator after cycling. Probably caused by uneven deposition of Zn.


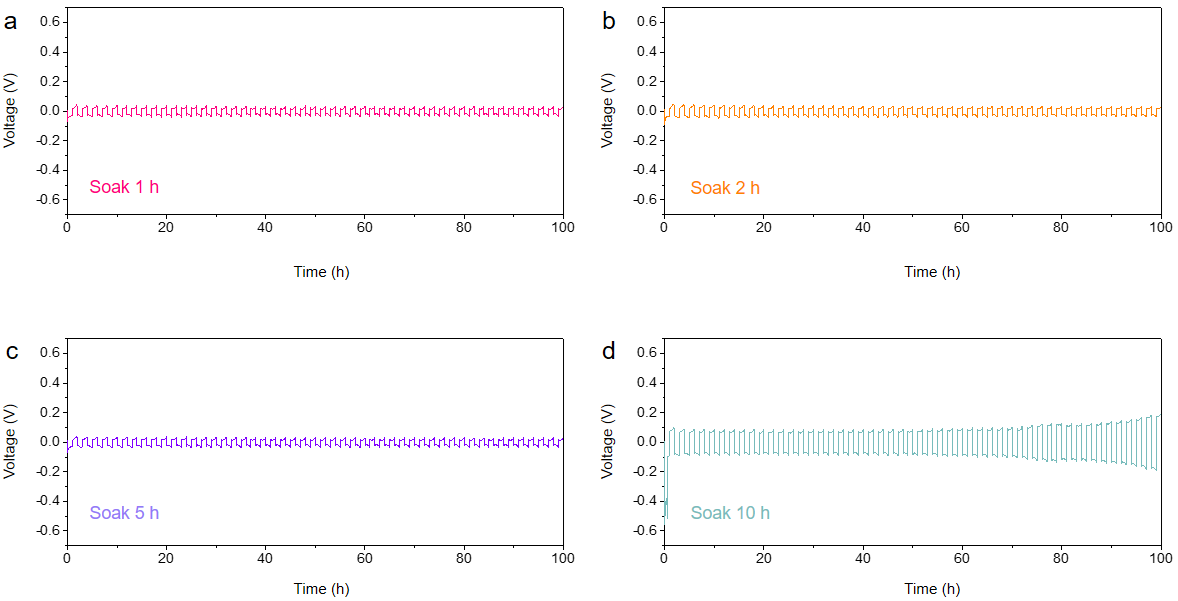


**Supplementary Fig. 8 | Voltage-time curves of Zn||Zn cells under 1 mA cm^−2^, 1 mAh cm^−2^.** The Zn electrodes were soaked in 1 m ZnSO_4_ + 0.1 m HTFSI electrolyte for different times before use. **a** soaking 1 h; **b** soaking 2 h; **c** soaking 5 h; **d** soaking 10 h.


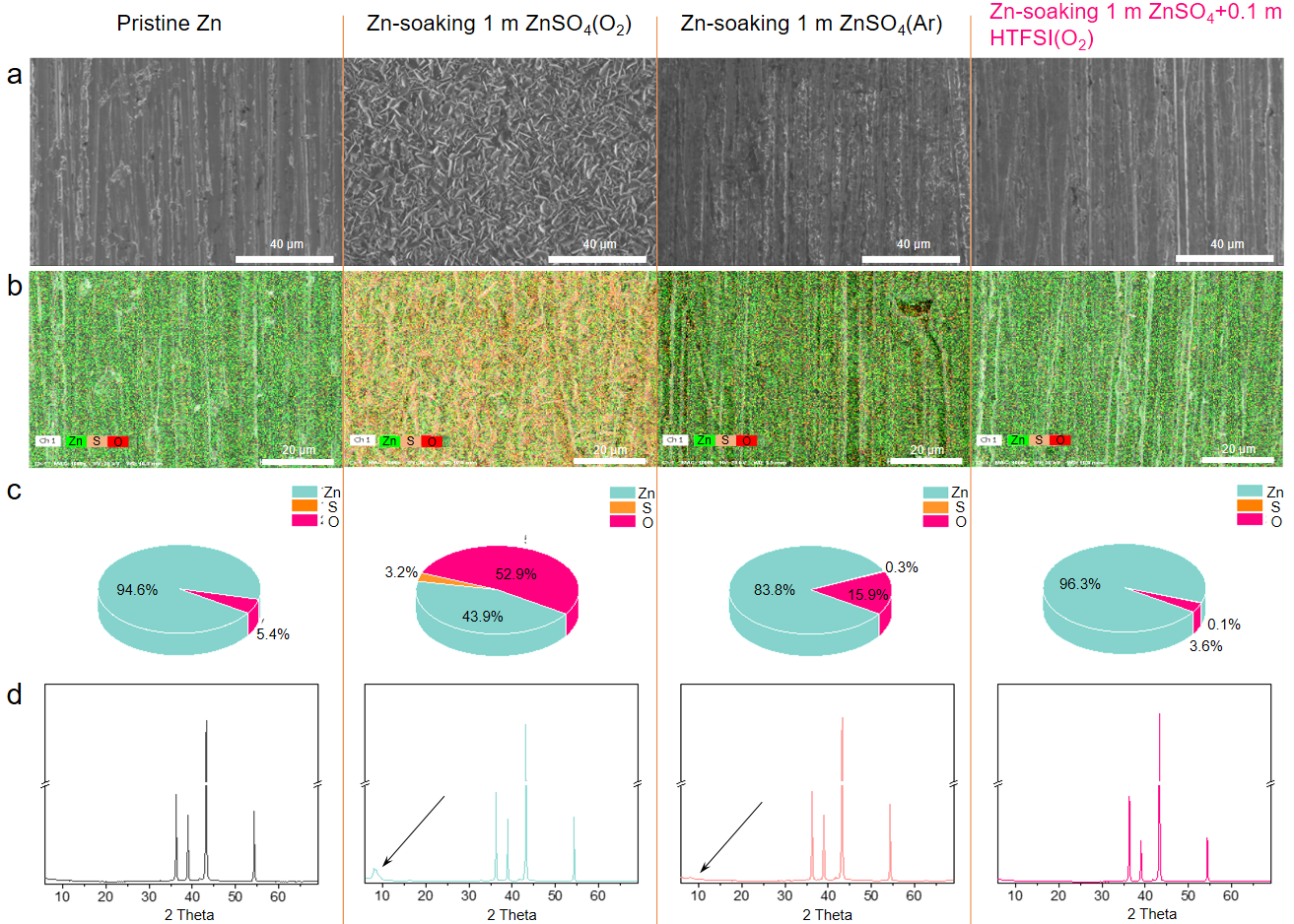


**Supplementary Fig. 9 | Effect of O_2_ on Zn Corrosion. a** SEM images **b** EDS elemental mapping **c** The atomic ratio column obtained from EDS elemental mapping **d** XRD patterns of pristine Zn and Zn electrodes surface after soak in 1 m ZnSO_4_ electrolytes (O_2_-saturated), 1 m ZnSO_4_ electrolytes (O_2_-free) and 1 m ZnSO_4_+0.1 m HTFSI electrolytes (O_2_- saturated) for 2 h.


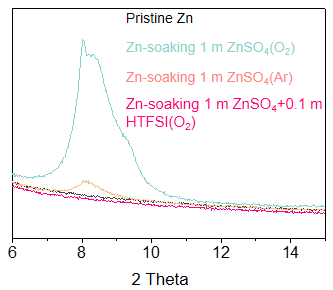


**Supplementary Fig. 10 | XRD characterization.** XRD pattern of pristine Zn and Zn electrodes surface after soaking in 1 m ZnSO_4_ electrolytes (O_2_-saturated), 1 m ZnSO_4_ electrolytes (O_2_-free) and 1 m ZnSO_4_+0.1 m HTFSI electrolytes (O_2_-saturated) for 2 h. pristine


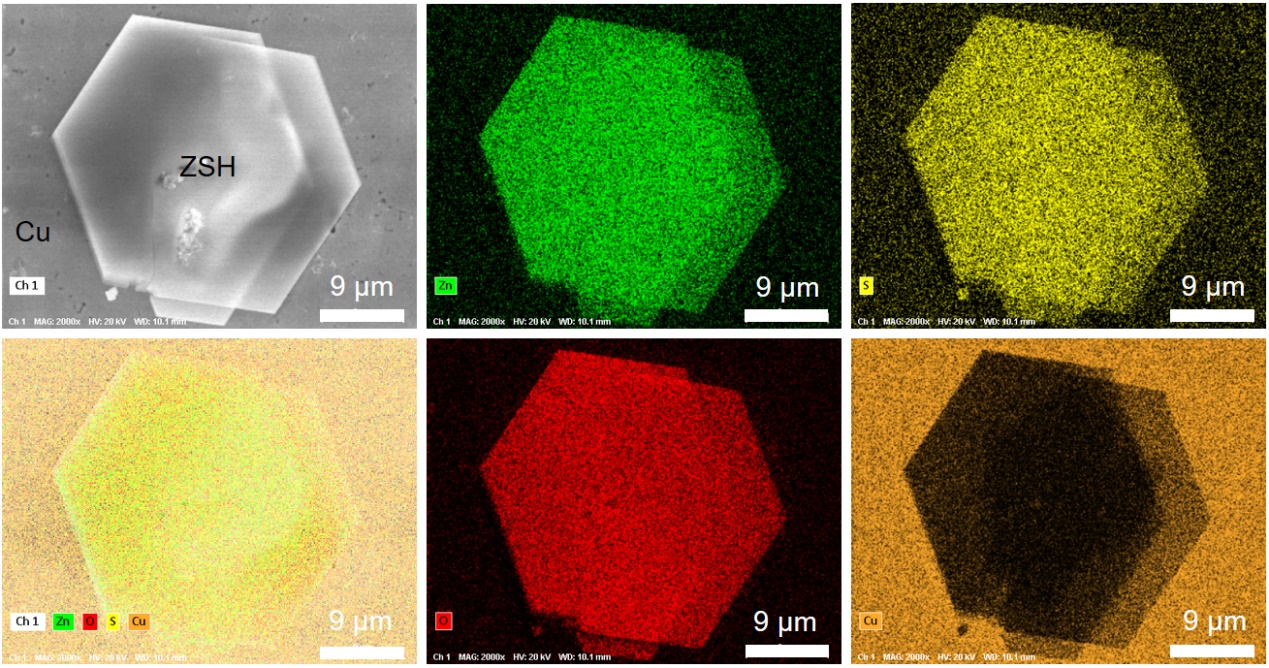


**Supplementary Fig. 11 | SEM and EDS characterizations** SEM image and EDS elemental mapping of Cu foil soaked in 1 m ZnSO_4_ electrolyte for 5 h.


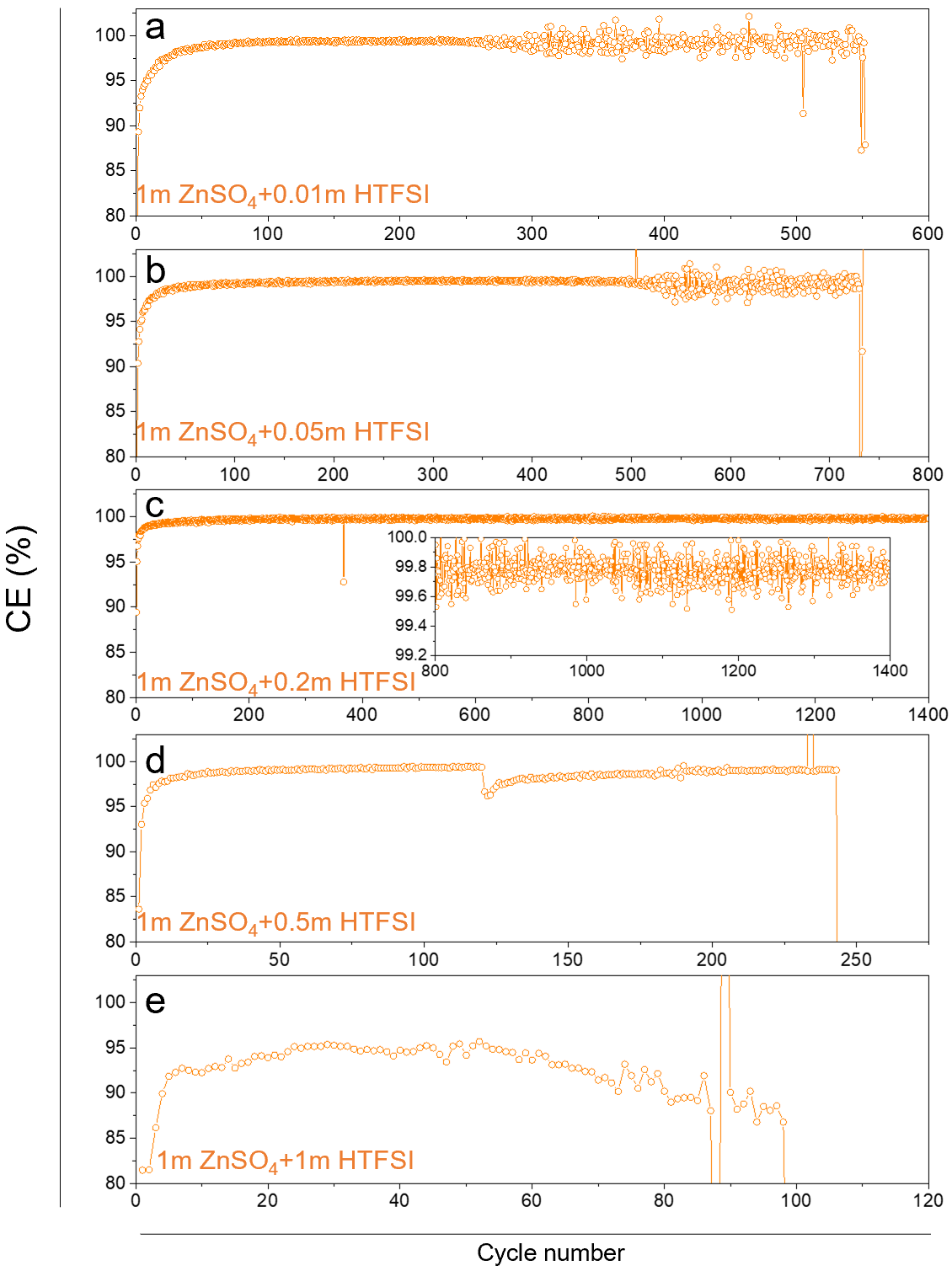


**Supplementary Fig. 12 | HTFSI concentration optimization.** Zn plating/stripping CE evolutions by cycling Zn||Cu cells in different electrolytes at 1 mA cm^−2^, 0.5 mAh cm^−2^.(**a**) 1 m ZnSO_4_+0.01 m HTFSI; (**b**) 1 m ZnSO_4_+0.05 m HTFSI; (**c**) 1 m ZnSO_4_+0.2 m HTFSI; (**d**) 1 m ZnSO_4_+0.5 m HTFSI; (**e**) 1 m ZnSO_4_+1 m HTFSI;

**
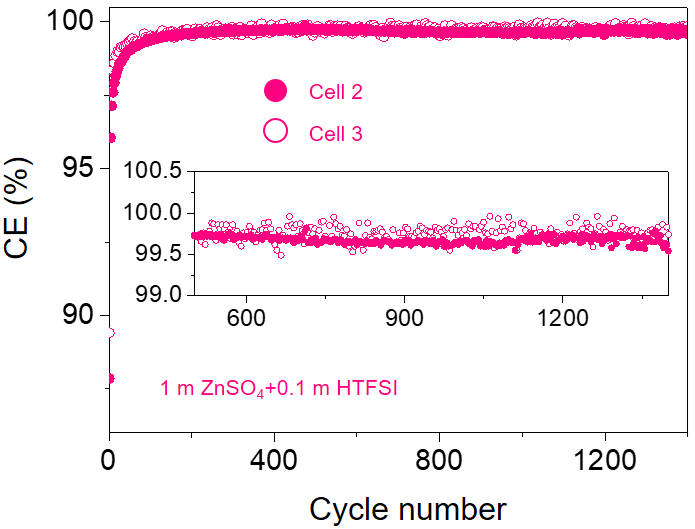
**

**Supplementary Fig. 13 | Parallel data of Coulumbic efficiency tests.** Zn CE evolution in Zn||Cu cells with 1 m ZnSO_4_+0.1 m HTFSI electrolytes at 1 mA cm^−2^, 0.5 mAh cm^−2^.


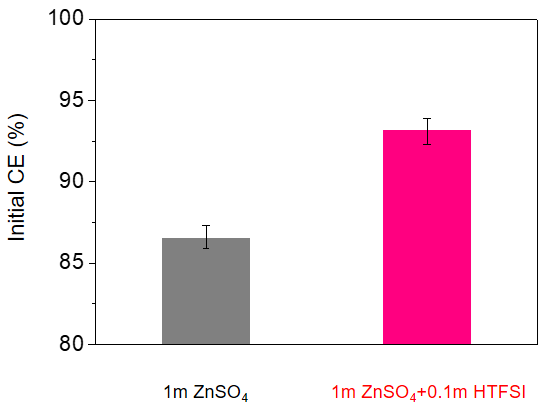


**Supplementary Fig. 14 | Zn initial CE in Zn||Cu cells with different electrolytes at 1 mA cm^−2^, 0.5 mAh cm^−2^.** Error bars correspond to the standard deviation of three independent measurements.


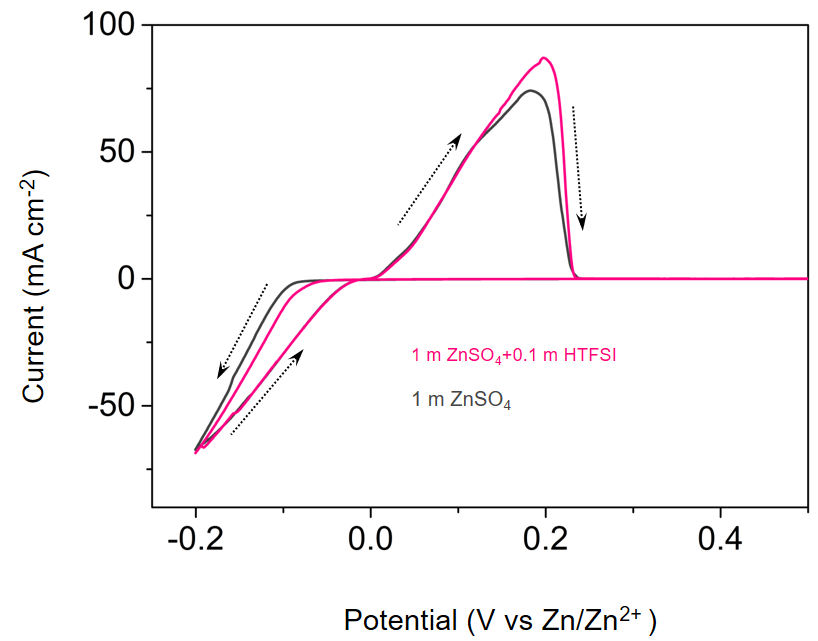


**Supplementary Fig. 15 | CV profiles of Zn plating/stripping in 1 m ZnSO_4_ and 1 m ZnSO_4_+0.1 m HTFSI electrolytes.** The Zn||Cu cells with a Scan speed of 5 mV s**^−^**^1^.

.


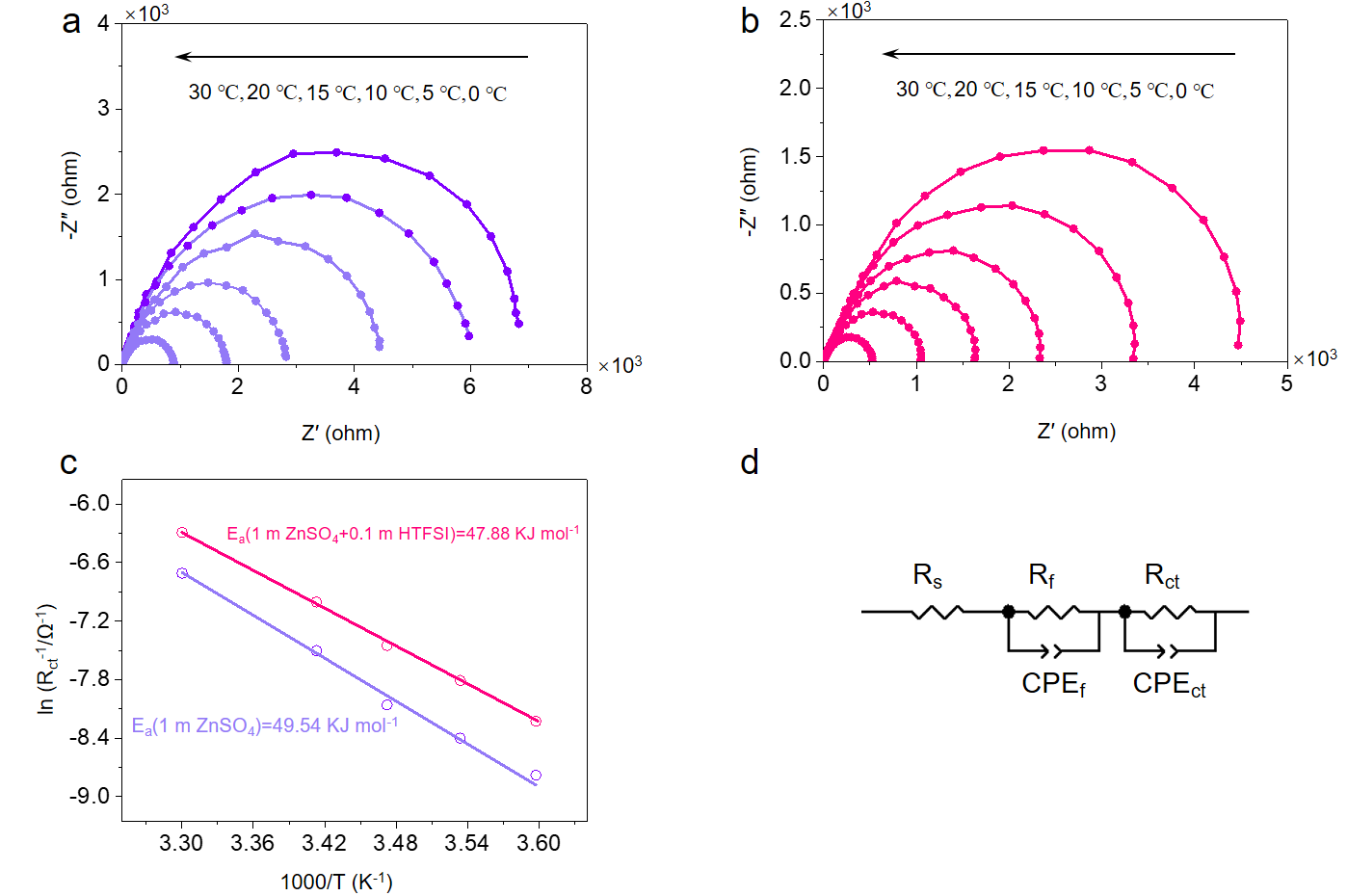


**Supplementary Fig. 16 | The Nyquist plots of Zn||Zn cells at different temperatures** in **a** 1 m ZnSO_4_ and **b** 1 m ZnSO_4_+0.1 m HTFSI electrolytes. **c** The Arrhenius behavior of temperature-dependent reciprocal resistances in 1 m ZnSO_4_ and 1 m ZnSO_4_+0.1 m HTFSI electrolytes. The charge transfer resistance (R_ct_) is obtained from the semicircle in the EIS curves. The activation energy was obtained by linear fitting ln(1/R_ct_) versus 1000/T in 1 m ZnSO_4_ and 1 m ZnSO_4_+0.1 m HTFSI electrolytes. **d** The equivalent circuit used for fitting the experimental EIS data.


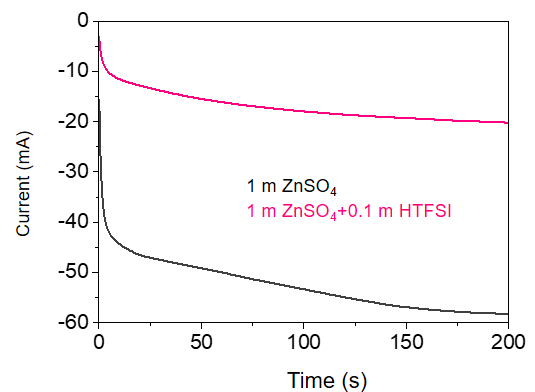


**Supplementary Fig. 17 | Zn deposition under constant voltage.** Chronoamperometric curves of Zn||Zn cells in 1 m ZnSO_4_ and 1 m ZnSO_4_+0.1 m HTFSI electrolytes at an overpotential of −150 mV.


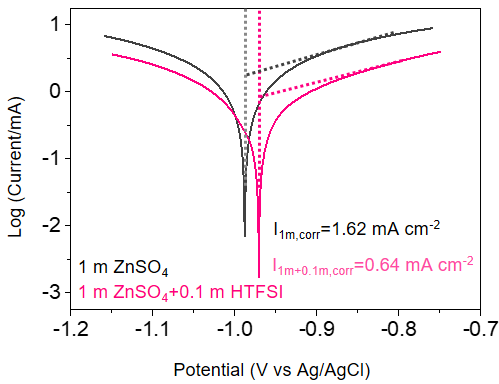


**Supplementary Fig. 18 | Linear polarization curves of Zn anodes.** Tests were done in 1 m ZnSO_4_ and 1 m ZnSO_4_+0.1 m HTFSI electrolytes. Zn metal is used as the working electrode and counter electrode, and Ag/AgCl electrode is the reference electrode.


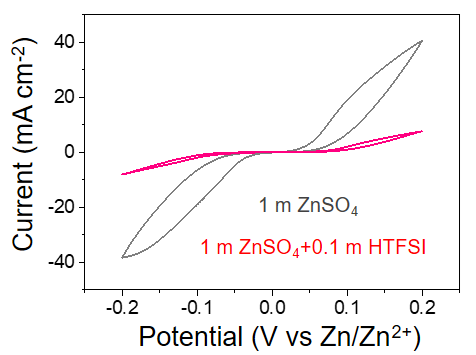


**Supplementary Fig. 19 | CV curves of Zn||Zn symmetrical cells with a scan speed of 5 mV s^−1^.**

The CV scan results of Zn||Zn cells in the two electrolytes exhibit noticeable distinctions, particularly in the response currents. The addition of HTFSI leads to a reduction in the response current. This likely indicates a smaller reaction surface area during the electrochemical process, which is consistent with the more uniform and dense deposition of Zn metal^1^.

**
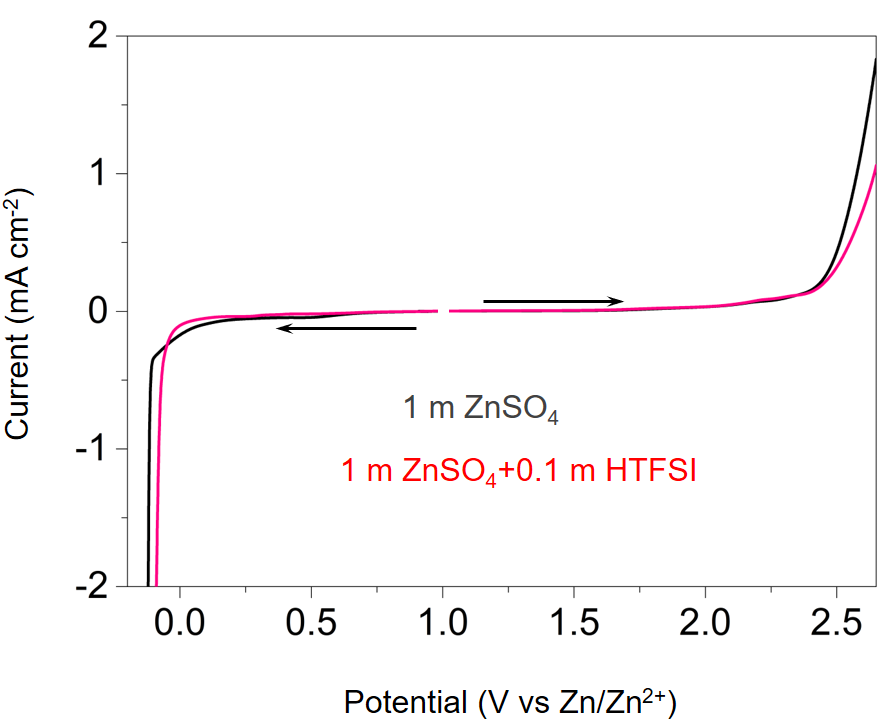
**

**Supplementary Fig. 20 | Electrochemical stability window of electrolyte with a scan speed of 5 mV s^−1^.**

To prevent the oxidation of Cu foil during the anodic scan, we assembled Zn||Ti cells for electrochemical stability window tests. It's noteworthy that distinguishing the hydrogen evolution potential and Zn deposition potential in aqueous electrolytes containing Zn salts can be challenging. Typically, sodium or lithium salts are used instead of Zn salts in electrochemical stability window tests. However, considering that the SEI layer on the Zn surface in the 1 m ZnSO_4_+0.1 m HTFSI electrolyte is indispensable for inhibiting hydrogen evolution, we did not replace the Zn salt in this case. As shown in Supplementary Fig. 19, in comparison to the 1 m ZnSO_4_ electrolyte, the oxygen evolution onset potential was increased in the 1 m ZnSO_4_+0.1 m HTFSI electrolyte, potentially due to the hydrophobic interface induced by TFSI^–^ anions. During the cathodic scan, it is difficult to differentiate the hydrogen evolution reaction and the Zn deposition reaction. However, on closer inspection, the initial increase of the cathodic current was delayed in the ZnSO_4_+0.1 m HTFSI electrolyte, which is possibly due to the formation of the SEI.


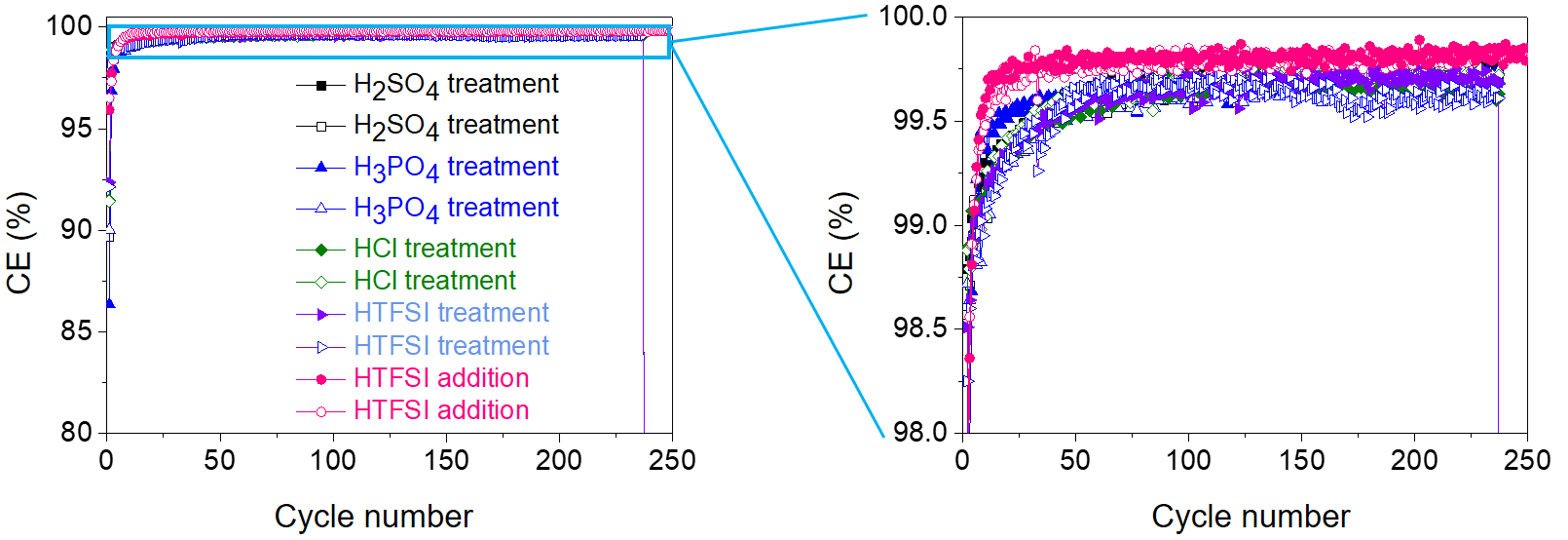


**Supplementary Fig. 21 | Comparison of Zn deposition/stripping properties of acid-treated Zn foil and acid-added electrolyte.** Zn||Cu cells operate at 1 mA cm^−2^, 0.5 mAh cm^−2^. Treat the Zn foil with 0.1 M acid for 10 minutes, then clean the Zn foil 3 times with acetone and water.


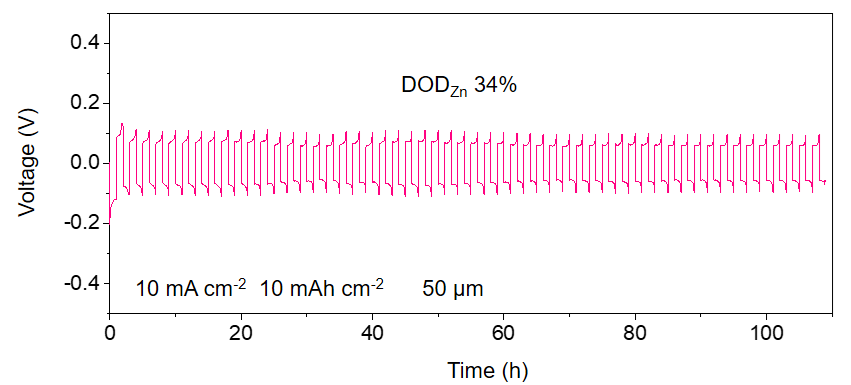


**Supplementary Fig. 22 | High Zn utilization test.** The cyclic stability of Zn||Zn cells with HTFSI additive under a DOD_zn_ of 34% with 10 mA cm^−2^; 10 mAh cm^−2^.


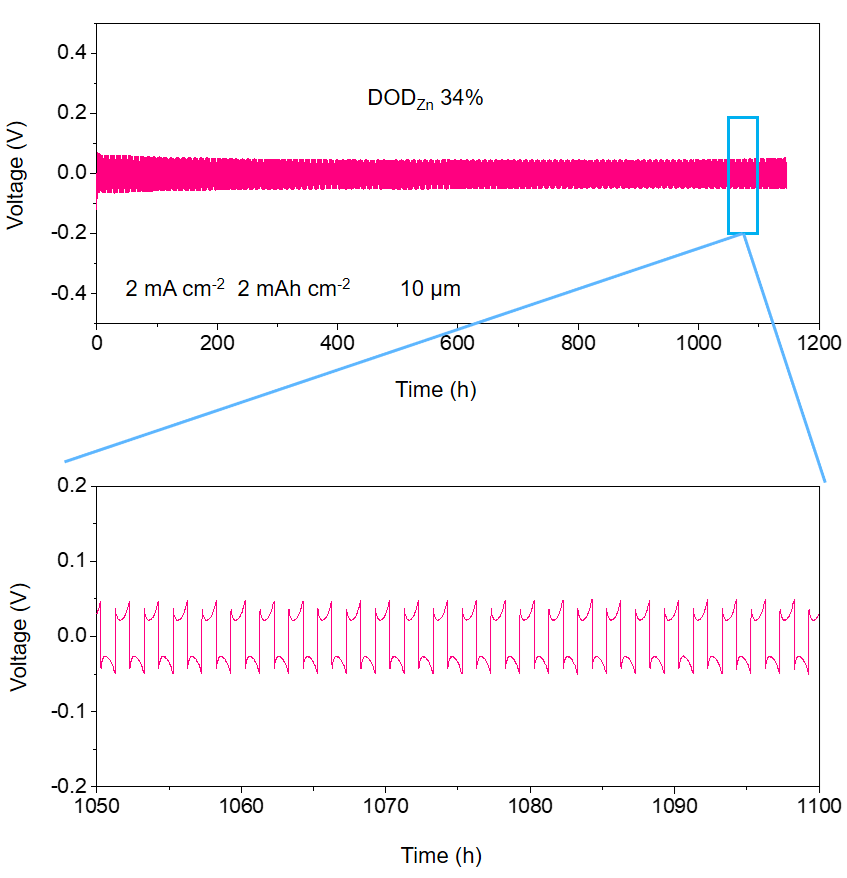


**Supplementary Fig. 23 | High Zn utilization test.** The cyclic stability of Zn||Zn (10 µm thickness) cells with HTFSI additive under a DOD of 34% with 2 mA cm^−2^; 2 mAh cm^−2^.


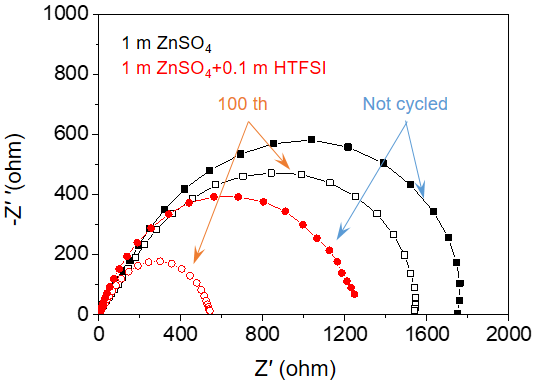


**Supplementary Fig. 24** | **Impedance tests of Zn||Zn cells.** The impedance spectra of the Zn||Zn cells in 1 m ZnSO_4_ and 1 m ZnSO_4_+0.1 m HTFSI electrolytes before and after cycling (100th cycle).


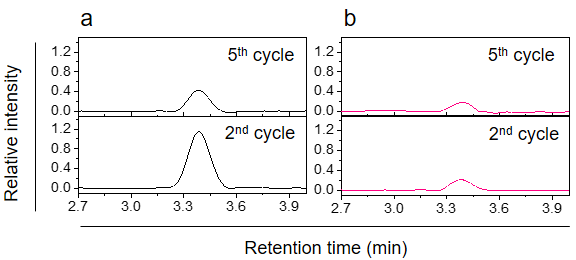


**Supplementary Fig. 25 | Comparison of gas production in two electrolytes.** Gas chromatography (GC) curves to test the H_2_ evolution in different aqueous media after the 2nd and 5th cycles. **a** 1 m ZnSO_4_; **b** 1 m ZnSO_4_+0.1 m HTFSI.


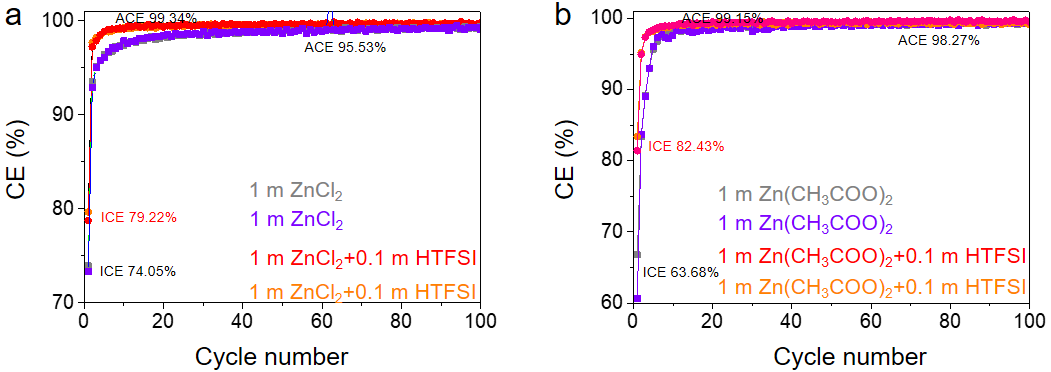


**Supplementary Fig. 26 | Zn CE evolution in Zn||Cu cells with electrolytes based on different salts.**  Zn||Cu cells operate at 1 mA cm^−2^, 0.5 mAh cm^−2^.


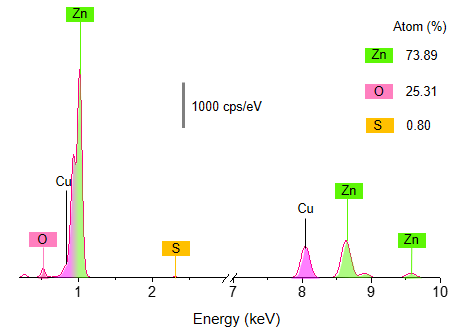


**Supplementary Fig. 27 | EDS characterization.** EDS pattern of plated Zn on Cu electrode in 1 m ZnSO_4_+0.1 m HTFSI electrolyte.


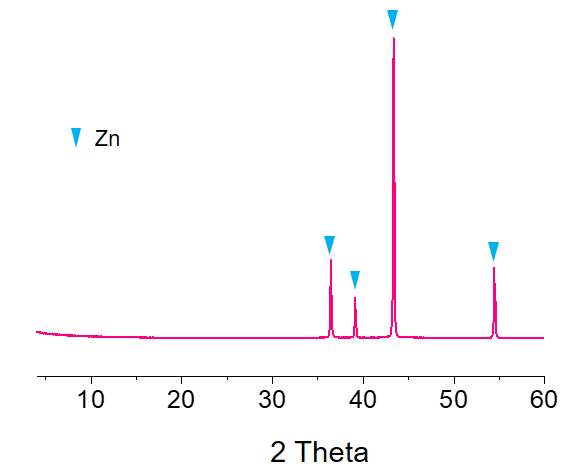


**Supplementary Fig. 28 | XRD characterization.** XRD pattern of plated Zn on Cu electrode in 1 m ZnSO_4_+0.1 m HTFSI electrolyte.


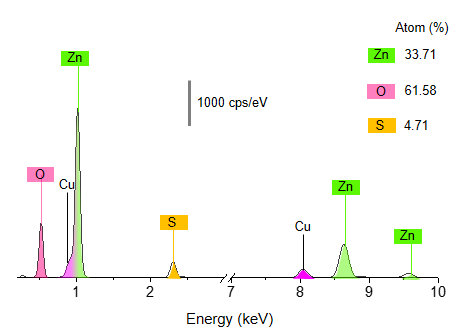


**Supplementary Fig. 29 | EDS characterization.** EDS pattern of plated Zn on Cu electrode in 1 m ZnSO_4_ electrolytes.


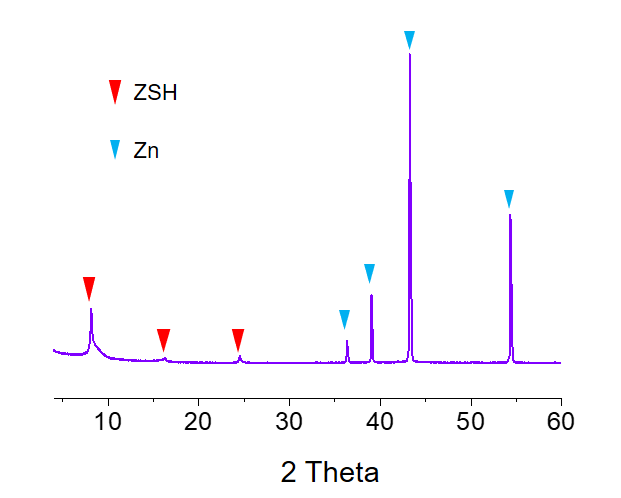


**Supplementary Fig. 30| XRD characterization.** XRD pattern of plated Zn on Cu electrode in 1 m ZnSO_4_ electrolyte.


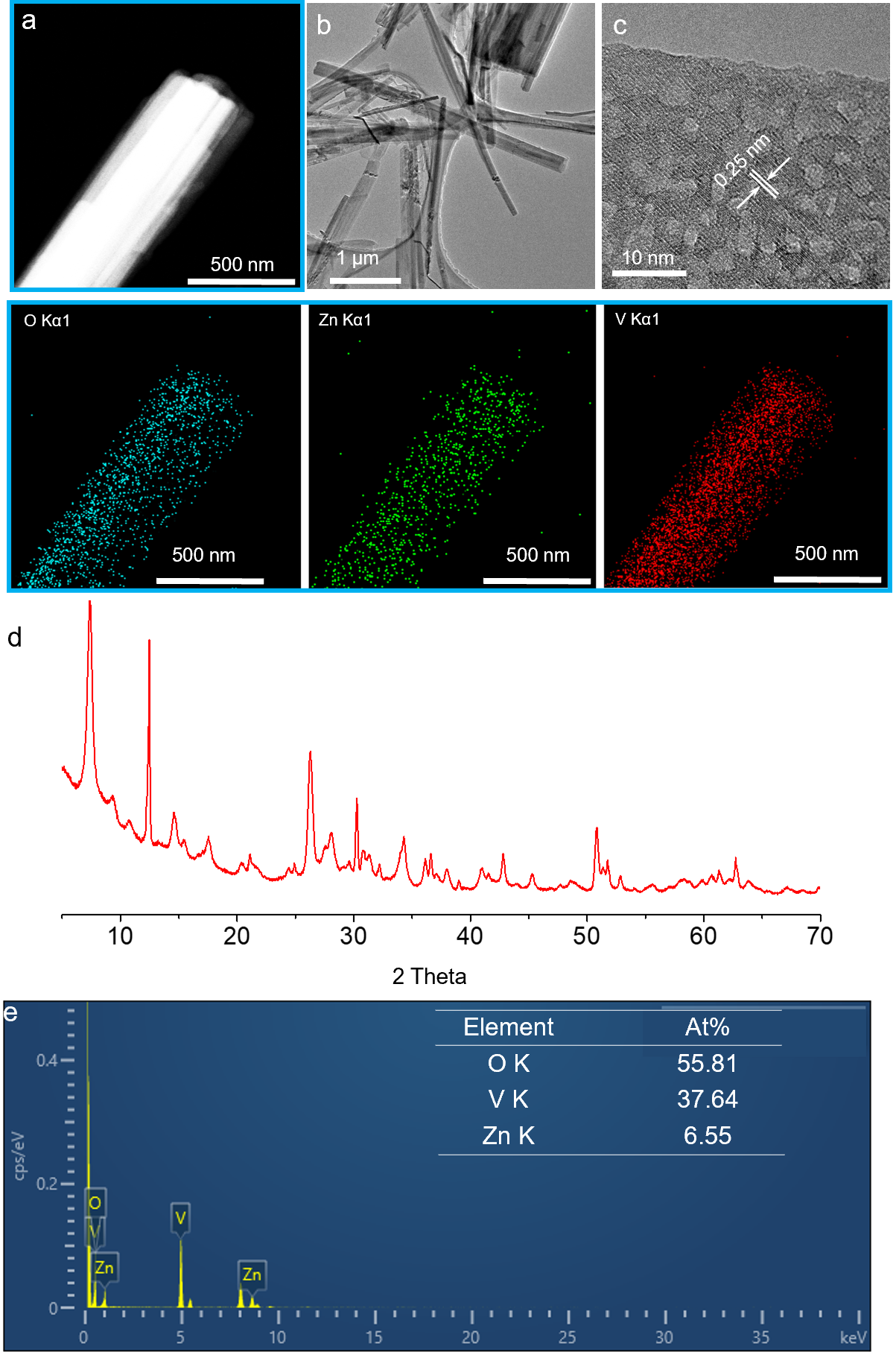


**Supplementary Fig. 31 | Characterization of ZVO cathode. a** TEM elemental mapping; **b** TEM; **c** high-resolution TEM images of ZVO; **d** XRD pattern of ZVO nanobelts; **e** TEM-EDS spectra of ZVO.

The electrochemistry of the 1 m ZnSO_4_+0.1 m HTFSI electrolyte was studied in a coin-type cell configuration at room temperature, using a ZnV_6_O_9_ (ZVO) cathode. The reason for choosing ZVO was because it eliminates the need for the activation step. In α-V_2_O_5_-based AZIBs, long activation times are usually required, making it impractical for practical use. To avoid this, we used a simple sol-gel method to synthesize ZVO and used it as the cathode in their AZIBs. The nanobelt morphology of ZVO was found to be homogeneous, as shown in Supplementary Fig. 29. The uniform distribution of Zn, V, and O in the ZVO nanobelts was further confirmed by TEM elemental mapping images in the same figure. Finally, the XRD pattern of the as-synthesized ZVO is presented in Supplementary Fig. 29d. The compositional characteristic of the ZVO electrode material is an atomic ratio of approximately 1:6:9 for Zn, V, and O, respectively (Supplementary Fig. 29e).


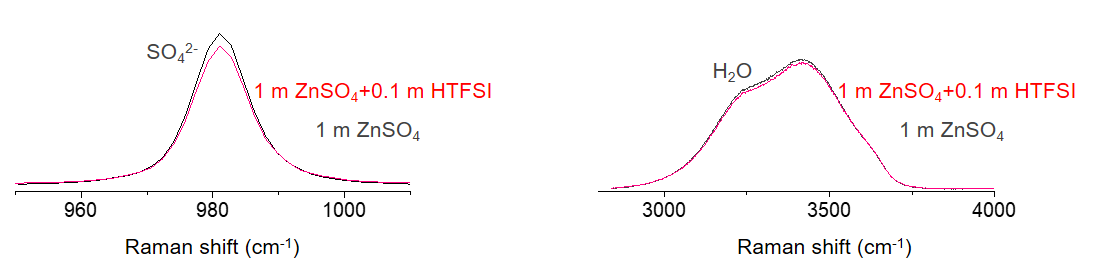


**Supplementary Fig. 32 | Raman spectra.** The Raman spectrum of 1 m ZnSO_4_+0.1 m HTFSI (red line) and 1 m ZnSO_4_ (black line) electrolyte.


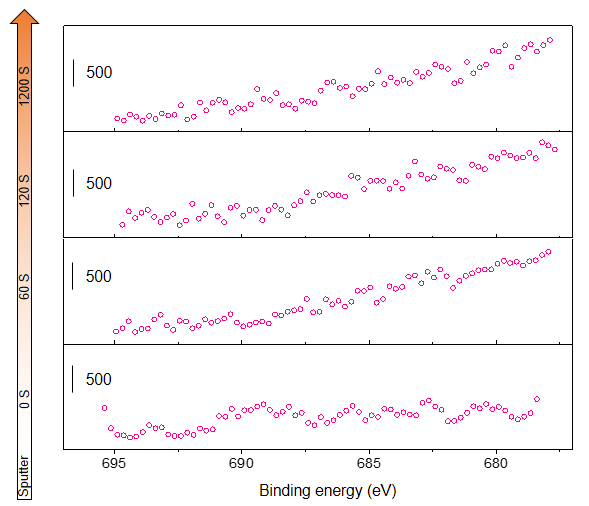


**Supplementary Fig. 33 | XPS characterization.** F *1s* XPS of Zn anode after soaking in 1 m ZnSO_4_+0.1 m HTFSI for 5 h.


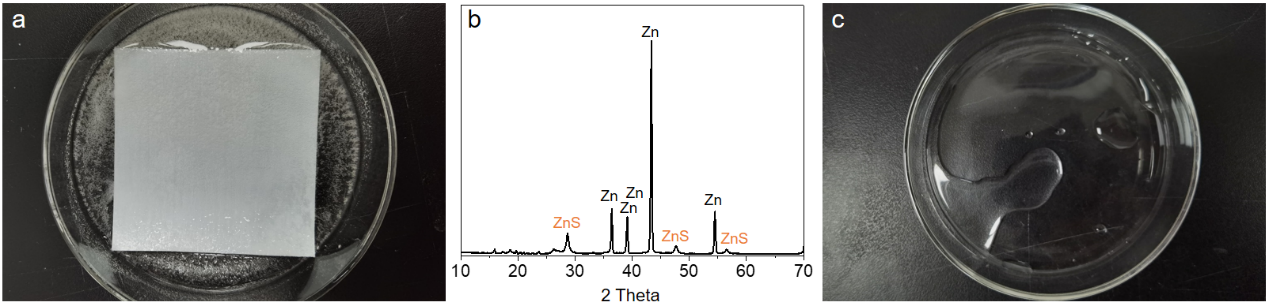


**Supplementary Fig. 34 | Characterization of ZnS formation.** The photograph **a** and XRD **b** of the HTFSI acid-1 m ZnSO_4_-Zn foil mixture and the photograph of the HTFSI-1 m ZnSO_4_ mixture **c**.


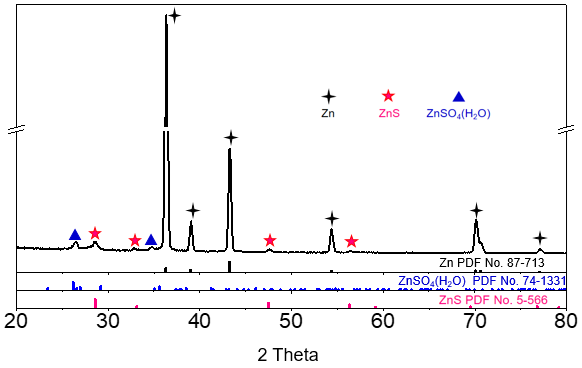


**Supplementary Fig. 35 |** **GIXRD characterization.** GIXRD pattern of the Zn anode after soaking in 1 m ZnSO_4_+0.1 m HTFSI for 5 h.


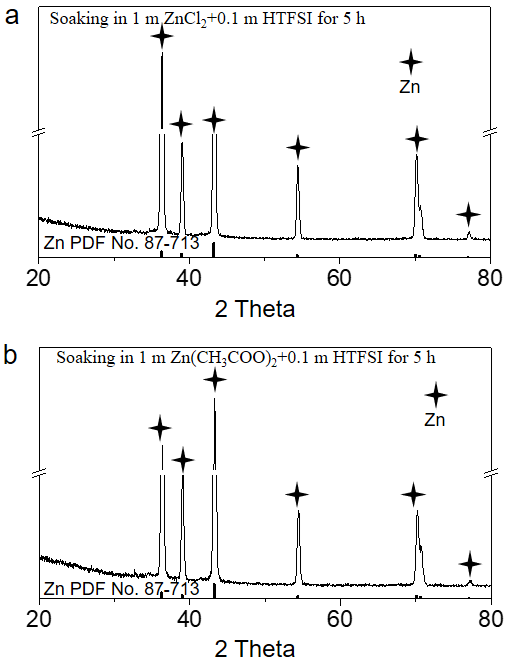


**Supplementary Fig. 36 | GIXRD characterization.** GIXRD patterns of the Zn anodes after soaking in 1 m ZnCl_2_+0.1 m HTFSI (a) and 1 m Zn(CH_3_COO)_2_+0.1 m HTFSI (b) for 5 h.


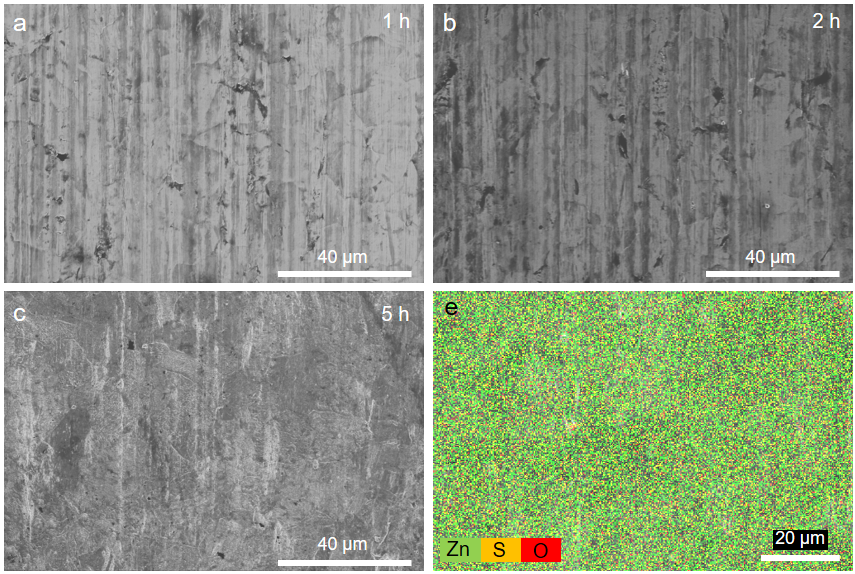


**Supplementary Fig. 37 | SEM and EDS characterizations.** SEM images of the surface of Zn electrodes after soaking in 1 m ZnSO_4_+0.05 m H_2_SO_4_ electrolytes for 1 h (**a**), 2 h (**b**), and 5 h (**c**); **d** EDS elemental mapping of Zn electrodes surface after soaking in 1 m ZnSO_4_+0.05 m H_2_SO_4_ electrolytes for 5 h.


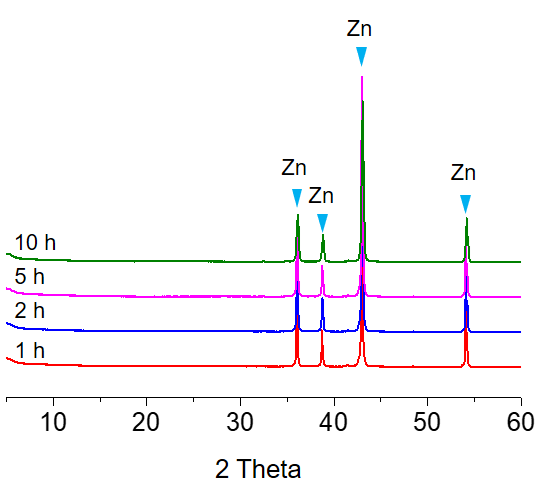


**Supplementary Fig. 38 | XRD characterization.** XRD patterns of Zn foil soaked in 1 m ZnSO_4_+0.05 m H_2_SO_4_ electrolytes for different times.


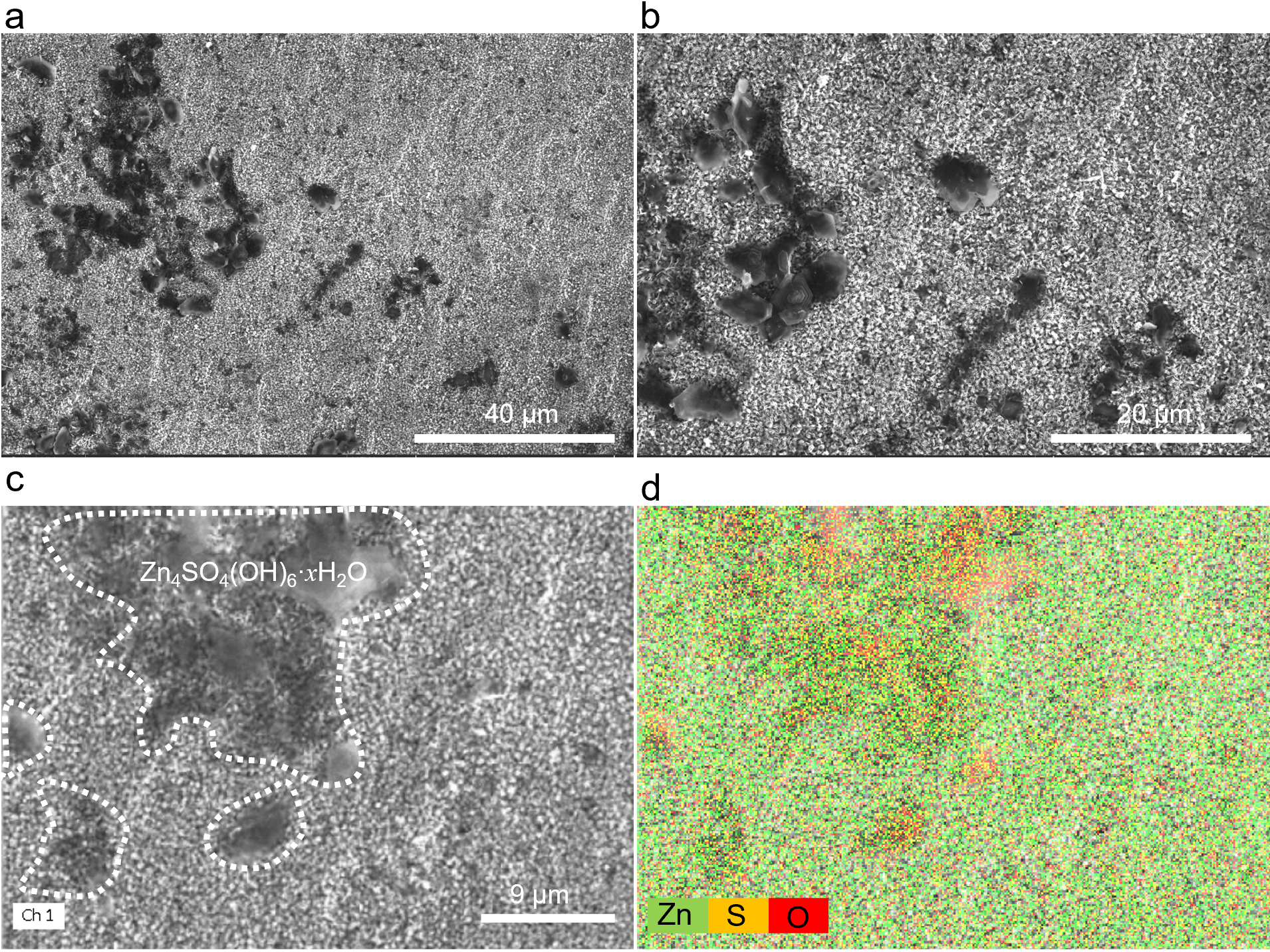


**Supplementary Fig.** **39 | SEM and EDS characterizations. a**, **b**, and **c** SEM images of plated Zn on Cu electrode in 1 m ZnSO_4_+0.05 m H_2_SO_4_ electrolyte; **d** EDS elemental mapping of Cu electrodes surface after plated Zn on Cu in 1 m ZnSO_4_+0.05 m H_2_SO_4_ electrolyte.


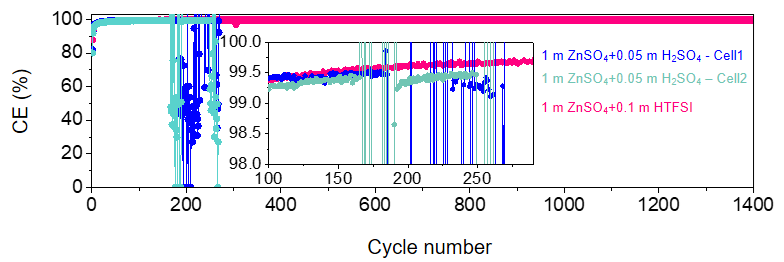


**Supplementary Fig.** **40 | Zn CE tests.** Comparative analysis of Zn CE evolution in 1 m ZnSO_4_+0.05 m H_2_SO_4_ electrolytes and 1 m ZnSO_4_+0.1 m HTFSI electrolytes. Zn||Cu cells operate at 1 mA cm^−2^, 0.5 mAh cm^−2^.


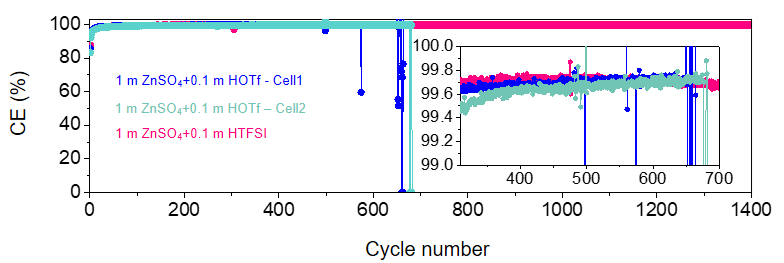


**Supplementary Fig. 41 |** **Zn CE tests.** Comparative analysis of Zn CE evolution in 1 m ZnSO_4_+0.1 m HOTf electrolytes and 1 m ZnSO_4_+0.1 m HTFSI electrolytes. Zn||Cu cells operate at 1 mA cm^−2^, 0.5 mAh cm^−2^.


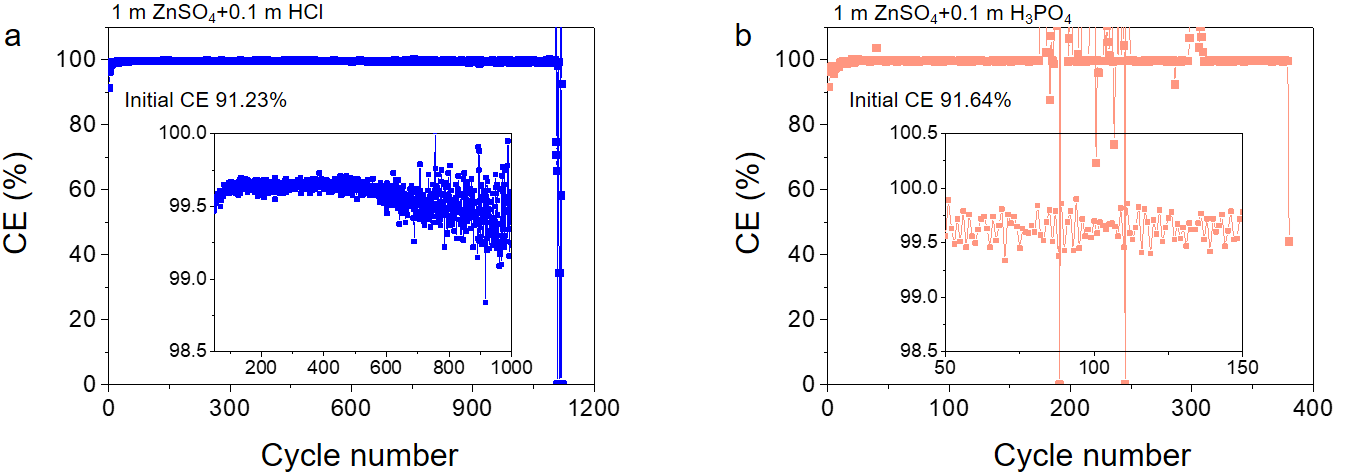


**Supplementary Fig. 42 | Zn CE evolution.** **a** 1 m ZnSO_4_+0.1 m HCl electrolytes; **b** 1 m ZnSO_4_+0.1 m H_3_PO_4_ electrolytes. Zn||Cu cells operate at 1 mA cm^−2^, 0.5 mAh cm^−2^.


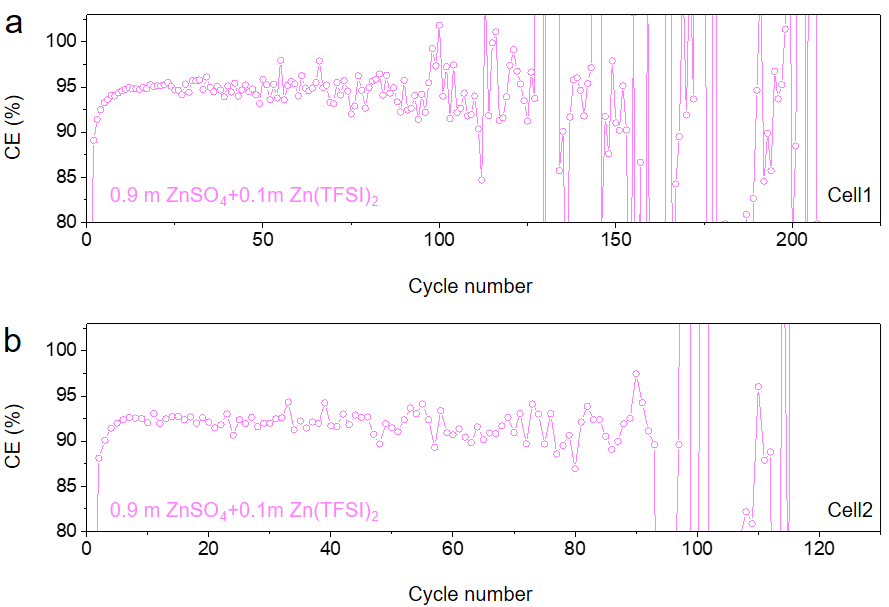


**Supplementary Fig. 43 | Zn CE evolution in Zn||Cu cells with 0.9 m ZnSO_4_+0.1 m Zn(TFSI)_2_ electrolytes.** **a** and **b** are parallel battery data. Zn||Cu cells operate at 1 mA cm^−2^, 0.5 mAh cm^−2^.


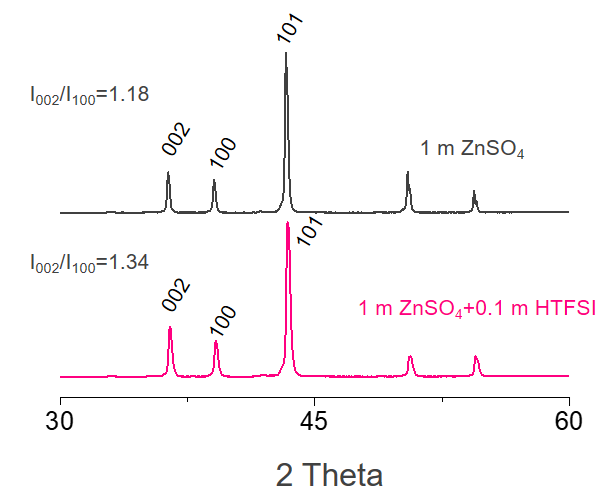


**Supplementary Fig. 44 | XRD characterization.** XRD of Zn deposited on Cu substrate at 1 mA cm^−2^, 10 mAh cm^−2^.


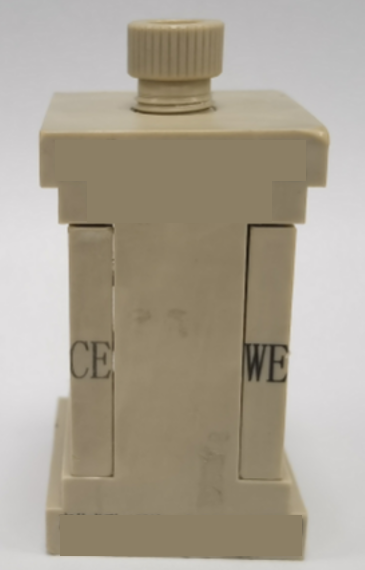


**Supplementary Fig. 45 | Photo of the mold used for ionic conductivity test.** The amount of electrolyte is about 1 mL, and the electrode area is 1 cm*1 cm.


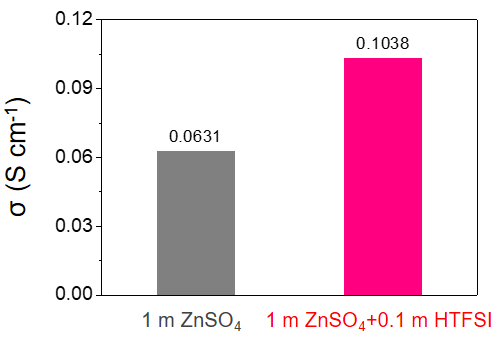


**Supplementary Fig. 46| Ionic conductivity of electrolytes.**


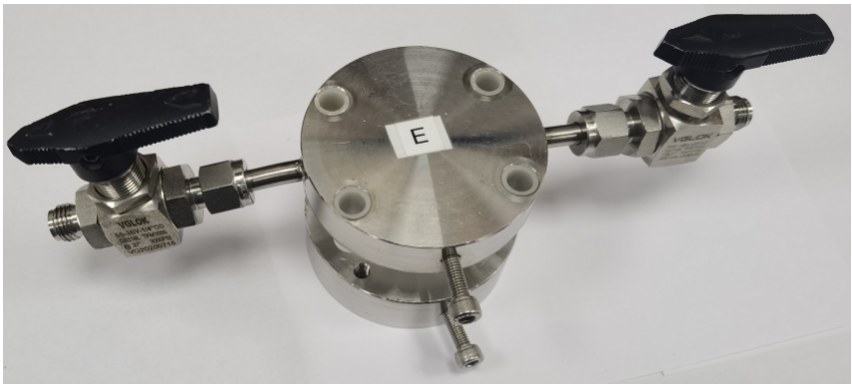


**Supplementary Fig. 47 | Photo of battery mold used for gas chromatography test.** Cycling test was first carried out with the mold, before purging the mold with argon for GC test.

**Supplemental Reference**

1 Zhao, K. *et al.* Boosting the Kinetics and Stability of Zn Anodes in Aqueous Electrolytes with Supramolecular Cyclodextrin Additives. *J. Am. Chem. Soc.* **144**, 11129-11137 (2022).
